# Histone H4 Lys 20 Monomethylation of the CENP-A Nucleosome Is Essential for Kinetochore Assembly

**DOI:** 10.1016/j.devcel.2014.05.001

**Published:** 2014-06-23

**Authors:** Tetsuya Hori, Wei-Hao Shang, Atsushi Toyoda, Sadahiko Misu, Norikazu Monma, Kazuho Ikeo, Oscar Molina, Giulia Vargiu, Asao Fujiyama, Hiroshi Kimura, William C. Earnshaw, Tatsuo Fukagawa

**Affiliations:** 1 Department of Molecular Genetics, National Institute of Genetics and The Graduate University for Advanced Studies (SOKENDAI), Mishima, Shizuoka 411-8540, Japan; 2 Comparative Genomics Laboratory, National Institute of Genetics, Mishima, Shizuoka 411-8540, Japan; 3 Cell Innovation Project, National Institute of Genetics, Mishima, Shizuoka 411-8540, Japan; 4 Wellcome Trust Centre for Cell Biology, University of Edinburgh, King’s Buildings, Mayfield Road, Edinburgh EH9 3JR, UK; 5 National Institute of Informatics, Hitotsubashi, Chiyoda-ku, Tokyo 101-8430, Japan; 6 Graduate School of Frontier Biosciences, Osaka University, 1-3 Yamada-oka, Suita, Osaka 565-0871, Japan

## Abstract

In vertebrate cells, centromeres are specified epigenetically through the deposition of the centromere-specific histone CENP-A. Following CENP-A deposition, additional proteins are assembled on centromeric chromatin. However, it remains unknown whether additional epigenetic features of centromeric chromatin are required for kinetochore assembly. Here, we used ChIP-seq analysis to examine centromere-specific histone modifications at chicken centromeres, which lack highly repetitive sequences. We found that H4K20 monomethylation (H4K20me1) is enriched at centromeres. Immunofluorescence and biochemical analyses revealed that H4K20me1 is present at all centromeres in chicken and human cells. Based on immunoprecipitation data, H4K20me1 occurs primarily on the histone H4 that is assembled as part of the CENP-A nucleosome following deposition of CENP-A into centromeres. Targeting the H4K20me1-specific demethylase PHF8 to centromeres reduces the level of H4K20me1 at centromeres and results in kinetochore assembly defects. We conclude that H4K20me1 modification of CENP-A nucleosomes contributes to functional kinetochore assembly.

## Highlights

• H4K20 monomethylation is enriched at both chicken and human centromeres
• H4K20 monomethylation occurs primarily at the CENP-A-containing nucleosomes
• H4K20 monomethylation is essential for kinetochore assembly in chicken cells

## Graphical Abstract

**Figure:**
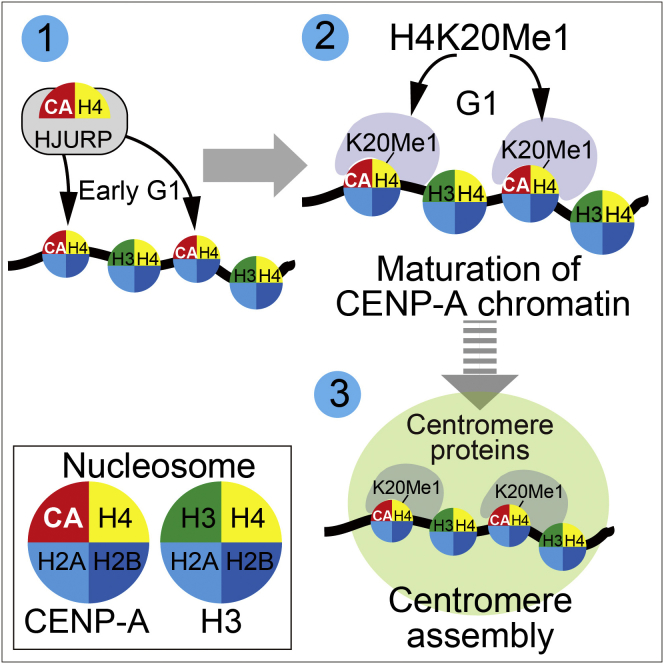

## Teaser

Deposition of histone CENP-A characterizes vertebrate centromeres, but the full epigenetic landscape of centromeres remains undefined. Hori et al. show that monomethylation of histone H4K20 is associated with CENP-A nucleosomes and is enriched at human and chicken centromeres. Importantly, they show that this chromatin modification is essential for kinetochore assembly.

## Introduction

Centromeres are essential genomic regions that direct faithful chromosome segregation. Despite their importance, centromeric DNA sequences are not evolutionally conserved (Allshire and Karpen, 2008), and studies of stable dicentric chromosomes and neocentromeres have revealed that centromeres are specified by sequence-independent epigenetic mechanisms in vertebrates (du Sart et al., 1997; Earnshaw and Migeon, 1985; Shang et al., 2013). The centromere-specific histone H3 variant CENP-A is a critical epigenetic marker for centromere specification (Allshire and Karpen, 2008; Guse et al., 2011; Hori et al., 2013; Mendiburo et al., 2011; Perpelescu and Fukagawa, 2011), but whether additional epigenetic features are required for centromere specification and/or kinetochore assembly remains a key unanswered question. In particular, it is unclear whether histone modifications (Ruthenburg et al., 2007) are required for distinct functions at centromeres.

Chromatin immunoprecipitation (ChIP) combined with massively parallel sequencing (ChIP-seq) provides a powerful approach for the genome-wide analysis of epigenetic modifications in vertebrate cells (Schones and Zhao, 2008). However, it is not possible to generate unambiguous maps of histone modification profiles across centromere regions in vertebrate cells because of the massively repetitive nature of the underlying centromeric and pericentromeric DNA sequences. Recent analyses of the chicken and horse genomes have revealed the presence of natural centromeres containing nonrepetitive DNA (Shang et al., 2010; Wade et al., 2009). In chicken, those nonrepetitive centromere sequences span ∼40 kb on chromosomes Z, 5, and 27 (Shang et al., 2010). This size of the CENP-A domain was confirmed by our chromosome engineering approach, which allowed us to efficiently generate neocentromeres in chicken DT40 cells and enabled us to examine the chromatin structure of nonrepetitive regions before and after they acquire centromere function (Shang et al., 2013). A recent study has further confirmed that the functioning kinetochore of chicken cells contains ∼50 kb of DNA (Ribeiro et al., 2014).

For this study, we exploited the nonrepetitive nature of DT40 centromeres to identify centromere-specific histone modifications. We find that H4K20 monomethylation (H4K20me1) is enriched at centromeres in DT40 cells. Finally, we demonstrate that H4K20me1 modification of the centromeric nucleosomes contributes to functional kinetochore assembly.

## Results

### H4K20 Monomethylation Is Detected at Centromere Regions in DT40 and HeLa Cells Based on ChIP Analyses

We began by performing ChIP-seq analyses in chicken DT40 cells using specific monoclonal antibodies against a range of core histone modifications, including H3K4me1/me2/me3, H3K9me1/me2/me3, H3K27me1/me2/me3, H3K36me1/me2/me3, and H4K20me1/me2/me3 (Figure S1 available online). Most of these histone modifications did not display any significant accumulation at centromeres assembled on nonrepetitive sequences (Figures S1A and S1C), although some of them were detected at repetitive centromeres, presumably because of the recognition of the associated heterochromatin (Figures S1B and S1D). For example, H4K20me3, an established marker for pericentromeric heterochromatin (Jørgensen et al., 2013), or H4K20me2 was detected at repetitive centromeres in chicken cells (Figure S1D), but not at centromeres containing nonrepetitive unique sequences, such as centromere Z, which lacks heterochromatin (Shang et al., 2010) (Figure S1C).

**Figure 1:**
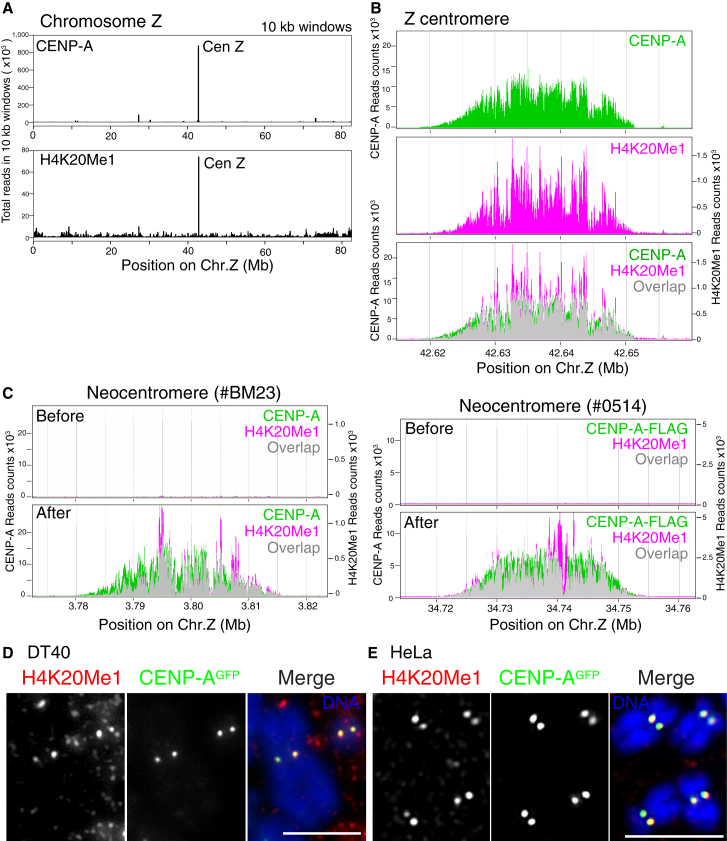
H4K20 Monomethylation Is Detected in Centromeres (A) ChIP-seq analysis with anti-CENP-A or anti-H4K20me1 antibodies on chromosome Z in DT40 cells. Sequence reads were mapped at 10 kb windows. Position of a major peak for H4K20me1 was identical to that for CENP-A. (B) High-resolution profile of ChIP-seq analysis for CENP-A (green) or H4K20me1 (magenta) around centromere region of chromosome Z (42.62–42.66 Mb region of chicken chromosome Z). Both profiles overlap (gray). (C) ChIP-seq analysis with anti-CENP-A (green) or anti-H4K20me1 (magenta) antibodies in neocentromere-containing DT40 cell lines (After #BM23 and #0514). ChIP-seq data at these neocentromere loci in parental Z3 cell line are also shown (before). (D) Immunofluorescence analysis with Cy3-labeled anti-H4K20me1 antibodies (red) in mitotic chromosomes in chicken DT40 cells expressing CENP-A-GFP (green). Colocalization of H4K20me1 with CENP-A was observed (merge). Scale bar, 5 μm. (E) Immunofluorescence analysis with Cy3-labeled anti-H4K20me1 antibodies (red) in mitotic chromosomes in human HeLa cells expressing CENP-A-GFP (green). Colocalization of H4K20me1 with CENP-A was observed (merge). Scale bar, 5 μm.

**Figure 2:**
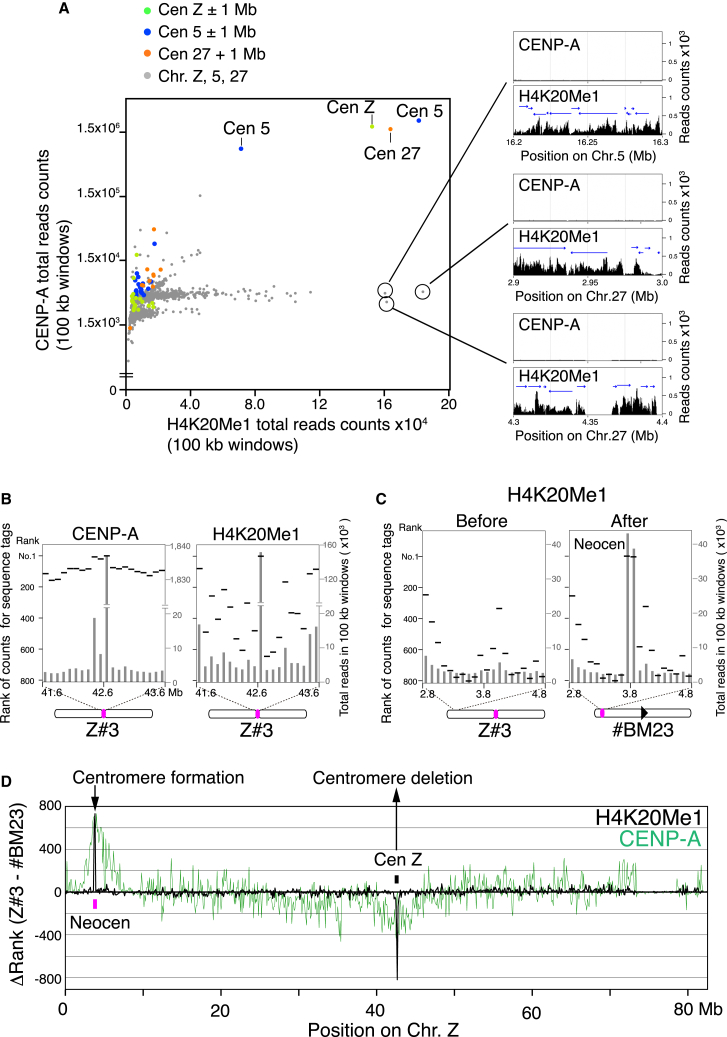
Coincidence of ChIP-Seq Peaks for CENP-A, and H4K20me1 Is Restricted in Centromere Regions (A) 2D plot for sequence reads in 100 kb region from chromosome Z, 5, and 27 by ChIP-seq with anti-CENP-A or anti-H4K20me1. Horizontal and vertical lines show sequence reads for H4K20me1 and CENP-A, respectively. Regions around centromeres 5, 27, and Z (±1 Mb) are colored green (Centromere Z), blue (Centromere 5), and orange (Centromere 27). H4K20me1-enriched regions in noncentromeres are marked by circles. Detailed ChIP-seq profiles in the regions marked by circles are also shown. Arrows shown in these ChIP-seq profiles indicate transcribed genes. (B) Counts of sequence reads (gray bar) for CENP-A- or H4K20me1-IP DNAs around centromere (Z#3) region. Ranking for these counts in chromosome Z are also shown (black line). (C) Counts of sequence reads (gray bar) for H4K20me1-IP DNAs around the neocentromere locus before (Z#3 cells) and after (#BM23 cells) neocentromere formation. Ranking for these counts in chromosome Z are also shown (black line). (D) Genome-wide ranking of numbers of sequence tags associated with CENP-A (green) or H4K20me1 (black) for Z#3 cells were subtracted from those for #BM23.

Strikingly, histone H4K20 monomethylation (H4K20me1) was highly enriched at both nonrepetitive (Figure 1A) and repetitive centromere regions (Figure S1D) in chicken DT40 cells. We confirmed this using the two independent monoclonal antibodies (15F11 and 22G3) against H4K20me1. Indeed, comparison of the ChIP-seq profile of H4K20me1 with that of CENP-A in the centromere of chromosome Z at high resolution, revealed that these profiles were largely coincident (Figure 1B). As expected, H4K20me1 was also present in noncentromere regions (Figure 1A), and we found an accumulation of H4K20me1 in the bodies of some transcribed genes (Figures 2A and S3A). These data are consistent with previous genome-wide analyses in human and mouse cells (Beck et al., 2012).

The analysis of neocentromere-containing cell lines allowed us to directly compare the chromatin modification status of specific genomic regions in the presence or absence of active centromere function. Such a comparison of the ChIP-seq profiles of H4K20me1 at loci before and after neocentromere formation in the cell lines #BM23 or #0514 (Shang et al., 2013) or the parental Z3 cell line is shown in Figure 1C. Prominent overlapping H4K20me1 and CENP-A peaks appeared following neocentromere formation (Figure 1C).

To examine whether H4K20me1 is also present in human centromeres, we performed ChIP-Southern analysis in human HeLa cells with anti-H4K20me1 antibodies for immunoprecipitation (IP) and α-satellite DNA as a probe for Southern hybridization. α-satellite DNA is a major DNA sequence of human centromeres (Allshire and Karpen, 2008). DNA precipitated with anti-H4K20me1 was hybridized with α-satellite DNA (Figure S1F), suggesting that H4K20me1 is also enriched in human centromeres.

Thus, based on ChIP-seq for chicken DT40 cells and ChIP-Southern for human HeLa cells, we conclude that H4K20me1 is enriched in centromeric chromatin in both chicken and human cells.

### H4K20 Monomethylation Occurs at All Centromere Regions in Chicken and Human Cells

We next performed immunofluorescence analysis using anti-H4K20me1 antibodies to directly visualize the centromeric accumulation of H4K20me1 in chicken and human cells. We stained either DT40 or HeLa cells expressing CENP-A-GFP with directly Cy3-labeled H4K20me1-antibodies and clearly detected colocalization of H4K20me1 with CENP-A-GFP signals in chicken and human cells (Figures 1D and 1E). In some cells, the signal/noise ratio was low, possibly because of steric hindrance of antibody binding. In addition, noncentromeric staining was also observed as expected, because H4K20me1 is located throughout the genome in transcribed regions. We present a gallery of representative immunofluorescence images in Figures S2A and S2C. We also confirmed the colocalization of H4K20me1 with CENP-A on chromatin fibers from DT40 cells (Figure S2B). Overall, we detected colocalization of H4K20me1 and CENP-A-GFP signals in more than 90% of interphase and mitotic cells.

Although we detected centromeric H4K20me1 signals in both interphase and mitotic cells, it is possible that levels of the modification might change across the cell cycle. To compare the levels of H4K20me1 at centromeres in DT40 cells, we prepared the chromatin fraction from both asynchronous and mitotic cell populations (Figure S2D) and performed immunoprecipitation with anti-CENP-A antibodies. We detected similar levels of H4K20me1 in CENP-A chromatin from asynchronous and mitotic populations (Figure S2E). ChIP-seq analysis also revealed similar H4K20me1 distributions at the Z centromere in asynchronous and mitotic populations of DT40 cells (Figure S2F). To determine whether the presence of H4K20me1 at centromeres is conserved across species, we also studied human (HeLa) cells (Figure S2G). Centromeric H4K20me1 levels were also constant across the cell cycle in HeLa cells (Figure S2H).

Based on this combination of immunofluorescence and biochemical data, we conclude that H4K20me1 occurs constitutively at all centromere regions in chicken and human cells.

### H4K20 Monomethylation in Noncentromeric Regions Does Not Induce Centromere Formation

H4K20me1 has been suggested to function in multiple chromatin transactions, including DNA replication, the DNA damage response, and transcription (Beck et al., 2012). Indeed, we confirmed that H4K20me1 is detected at multiple genomic loci based on ChIP-seq with chicken DT40 cells (Figures 2A and S3A), but the majority of those peaks did not display significant CENP-A accumulation (Figures 2A and S3A). We examined the correlation between the numbers of DNA sequence reads associated with CENP-A and H4K20me1 in chicken chromosome 5, 27, and Z, which contain nonrepetitive centromeres (Figure 2A). Importantly, we observed significant enrichment of both CENP-A and H4K20me1 (positive correlation) only at centromeres on those chromosomes (Figure 2A). Although we found some H4K20me1-enriched regions on the arms of chromosomes 5, 27, and Z (circles in Figure 2A), CENP-A accumulation was not detected there.

We also detected some ectopic CENP-A accumulation at noncentromeric loci (Figures 2B–2D) (Shang et al., 2013), but these regions did not display H4K20me1 enrichment (Figures 2B–2D). As shown in Figure 2B, a 2 Mb region surrounding the Z centromere region displayed a consistently high number of CENP-A-associated sequence tags (ectopic CENP-A “cloud”), but H4K20me1 enrichment was not detected in this region. In addition, H4K20me1 did not accumulate in the region surrounding the newly formed neocentromere in #BM23 cells (Figure 2C). We also compared the number of CENP-A- or H4K20me1-associated sequence tags of #BM23 cells (neocentromere-containing cells) with those of Z#3 (parental cells) by a genome-wide difference analysis (Figure 2D) and confirmed that H4K20me1 enrichment was not detected at regions flanking centromeres, where ectopic noncentromeric CENP-A was clustered. These analyses (Figure 2) suggest that H4K20me1 accumulation may not simply induce CENP-A incorporation in chicken DT40 cells.

To test this hypothesis in human cells experimentally, we tethered the catalytic domain of prSET7 fused to tetR-EYFP into an ectopic alphoid^tetO^ array inserted into a chromosome arm in HeLa cells (Figures S3B and S3C). prSET7 is the enzyme primarily responsible for monomethylation of H4K20 (Nishioka et al., 2002), and as expected, this tethering resulted in a significant accumulation of H4K20me1 on the alphoid^tetO^ array (Figures S3D and S3E). In those cells, CENP-A did not accumulate on the alphoid^tetO^ array (Figures S3F and S3G). Therefore, H4K20me1 is not sufficient to recruit CENP-A in human cells. In controls, targeting of tetR-EYFP-HJURP to the same array gave very high levels of CENP-A incorporation. We note that whereas prSET7 did induce H4K20me1 on the alphoid^tetO^ array, it is possible that H4K20me1 at centromere regions may be induced by another Set domain protein.

Together, these experiments demonstrate that significant coenrichment for both CENP-A and H4K20me1 is only observed at active centromeres.

### H4K20 Monomethylation Primarily Occurs at the CENP-A Nucleosomes

CENP-A nucleosomes are interspersed with histone H3-containing nucleosomes at centromeres in human and chicken cells (Hori et al., 2008; Sullivan and Karpen, 2004), and both types of nucleosomes appear to be critical for establishing centromeric chromatin (Hori et al., 2008). Interestingly, centromeric histone H3-containing nucleosomes have modifications that are associated with transcribed genes (Sullivan and Karpen, 2004), and these are required for centromere specification and kinetochore assembly (Bergmann et al., 2011). Therefore, it is essential to define whether H4K20me1 modifies CENP-A or H3 nucleosomes in centromeric regions. To test this, we performed IP with anti-FLAG or anti-CENP-A antibodies to isolate CENP-A-containing mononucleosomes from DT40 cells expressing CENP-A-FLAG or from HeLa cells.

**Figure 3:**
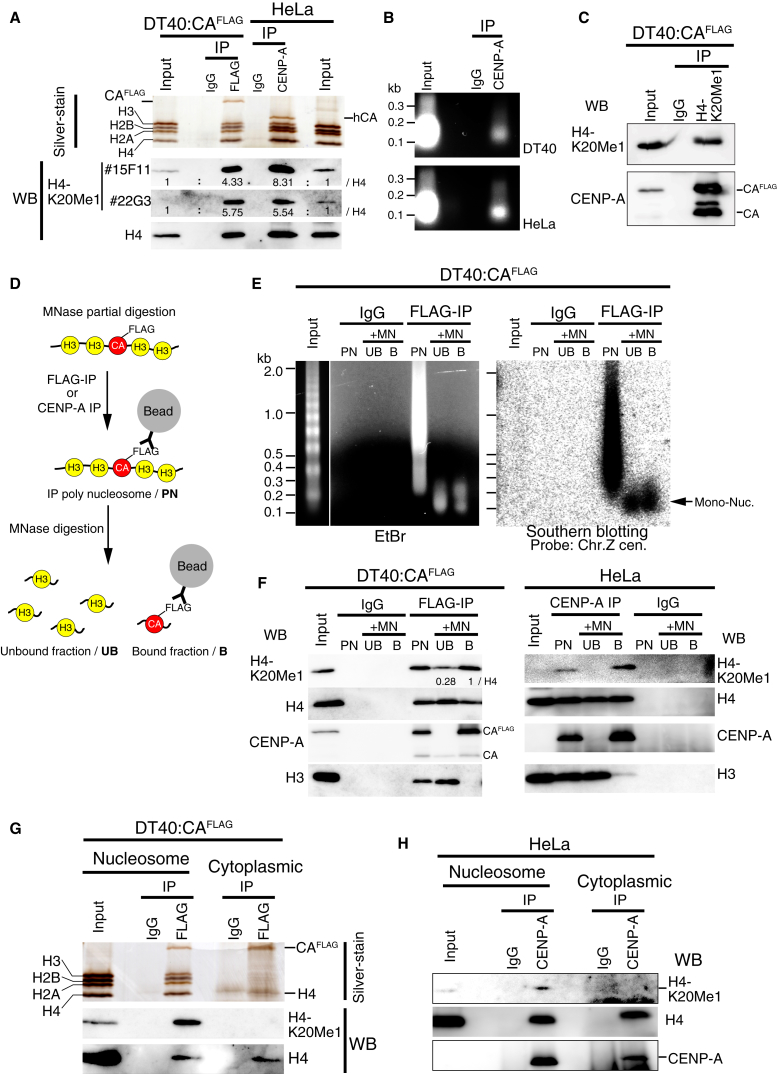
H4K20 Monomethylation Occurs at the CENP-A Nucleosomes (A) Chromatin was completely digested with MNase, and the mononucleosome fraction was isolated from HeLa or DT40 cells expressing CENP-A-FLAG (input). Immunoprecipitation (IP) experiments were performed with anti-human CENP-A (for HeLa) or FLAG (for DT40) antibodies, and the CENP-A nucleosomes were recovered. Western blot analysis was carried out for the CENP-A nucleosomes with two kinds of anti-H4K20me1 (15F11 or 22G3) and -panH4 antibodies. Silver-stained gel image is also shown. (B) Gel electrophoresis of DNAs extracted from the mononucleosome fractions used in (A). (C) IP experiments were performed with anti-H4K20me1 antibodies in the mononucleosome fraction from DT40 cells expressing CENP-A- FLAG, and western blot analysis was carried out with anti-H4K20me1 and -CENP-A antibodies. (D) An experimental design to prepare CENP-A nucleosome (B) and histone H3 nucleosome (UB) fractions in centromere region. Chromatin from HeLa or DT40 cells expressing CENP-A-FLAG was partially digested with MNase and performed IP with anti-human CENP-A (for HeLa) or FLAG (for DT40) antibodies. The precipitated fraction was completely digested with MNase again. The beads unbound supernatant fraction (UB) was expected to contain H3 nucleosome around CENP-A nucleosomes, and beads bound fraction (B) was expected to contain CENP-A mononucleosomes. (E) DNAs were recovered from UB or B fractions, and Southern blot analysis was performed with a centromeric DNA probe (CenZ). DNAs from both fractions were hybridized the CenZ probe. (F) Western blot analysis for UB or B fractions prepared from DT40 or HeLa cells with anti-H4K20me1, -pan H4, -CENP-A, and -H3 antibodies. In the UB fraction, H3, but not CENP-A, was detected, and CENP-A, but not H3, was detected in the B fraction. H4K20me1 was highly enriched in CENP-A-containing chromatin from both DT40 and HeLa cells. (G) Purification of the cytoplasmic CENP-A-H4 complex before deposition to nucleosome from DT40 cells expressing CENP-A-FLAG. Western blot analysis for the CENP-A-H4 complex with anti-H4K20me1 and -panH4 antibodies was performed. Silver-stained gel image is also shown. (H) Western blot analysis for the cytoplasmic CENP-A-H4 complex before deposition to nucleosome from HeLa cells with anti-H4K20me1, -panH4, and -CENP-A antibodies.

Western blotting analysis using two independent antibodies against H4K20me1 (15F11 and 22G3) revealed a strong enrichment of H4K20me1 in CENP-A nucleosomes from both cell types (Figure 3A). For this experiment, we digested the chromatin fraction extensively with MNase. We note that although a majority of this MNase-digested fraction contains mononucleosomes (Figure 3B), the fraction still contains some centromeric H3 nucleosomes because of incomplete digestion of CENP-A chromatin (Figure 3A). In reciprocal experiments, we found that CENP-A was enriched in immunoprecipitates generated using anti-H4K20me1 antibodies in DT40 cells (Figure 3C). CENP-A chromatin was significantly enriched in H4K20me1 IPs, suggesting that most CENP-A nucleosomes at centromeres are monomethylated at H4K20.

To test whether centromeric H3 nucleosomes adjacent to CENP-A nucleosomes are also modified by H4K20me1, we directly compared the level of H4K20me1 at CENP-A nucleosomes with that on the H3 nucleosomes present at centromeres (Figure 3D). To do this, we partially digested chromatin of cells expressing CENP-A-FLAG with MNase and performed IPs with anti-FLAG antibodies (polynucleosome: PN in Figure 3D). We then redigested the PN fraction with MNase and then collected bead-bound and bead-unbound fractions (Figure 3D). We confirmed that both fractions contained centromere DNA based on Southern hybridization (Figure 3E), indicating that nucleosomes from both fractions are centromeric. Through this fractionation, we could isolate fractions consisting of CENP-A nucleosomes (bead-bound, B) and centromeric H3 nucleosomes (bead-unbound, UB).

In the bead-bound fraction, only CENP-A was detected. In contrast, histone H3, but not CENP-A, was detected in the unbound fraction (Figure 3F). This fractionation suggests that most CENP-A nucleosomes in both chicken and human cells are homotypic (i.e., CENP-A/CENP-A). Although Lacoste et al. (2014) reported CENP-A/H3.3 heterotypic nucleosomes at noncentromere regions, such nucleosomes may occur only infrequently in centromeres. H4K20me1 was detected in both fractions but was enriched in the CENP-A nucleosome fraction compared with the H3 nucleosome fraction in both chicken and human cells (B versus UB fractions in Figure 3F). Thus, H4K20me1 occurs primarily on CENP-A nucleosomes.

If H4K20me1 modifies histone H4 present in CENP-A nucleosomes, it is possible that this methylation occurs to the CENP-A-H4 complex prior to the deposition of CENP-A at centromeres. To test this, we purified CENP-A-H4 complexes from the cytoplasmic (nonincorporated) fraction from chicken and human cells and performed western blotting analysis using anti-H4K20me1 antibodies. We did not detect H4K20me1 in the cytoplasmic CENP-A-H4 complex from either chicken or human cells (Figures 3G and 3H), suggesting that the H4K20me1 modification occurs following CENP-A deposition into centromeric chromatin.

In summary, we conclude that centromeric H4K20me1 occurs primarily on CENP-A-containing nucleosomes following CENP-A deposition into centromeres of both chicken and human cells.

### H4K20 Monomethylation at Centromeres Is Essential for Kinetochore Assembly

**Figure 4:**
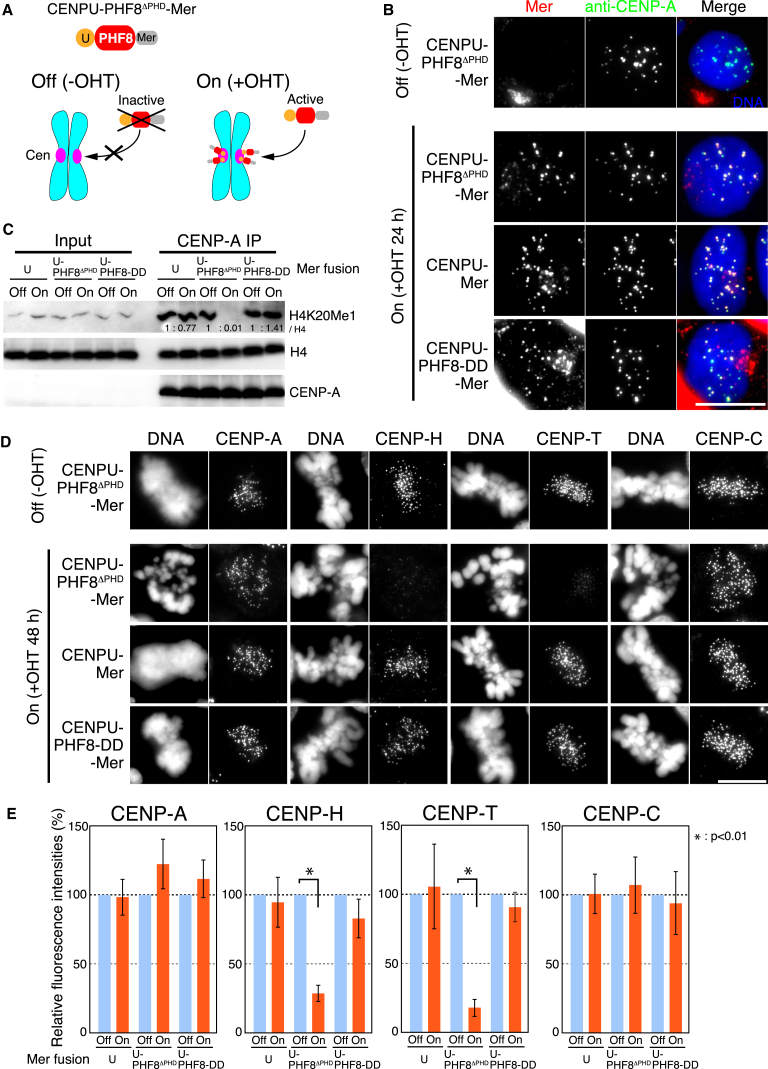
Reduction of H4K20 Monomethylation at Centromeres Causes Defects of Kinetochore Assembly (A) An experimental strategy for localization of CENP-U-PHF8 fusion into centromere region using DT40 cells. Catalytic domain (Jumonji) of PHF8 (PHF8^ΔPHD^) was fused with CENP-U and a murine estrogen receptor (Mer) tag, and the CENP-U-PHF8^ΔPHD^-Mer fusion construct was introduced into CENP-U-deficient DT40 cells. The CENP-U-PHF8^ΔPHD^-Mer fusion protein is activated by addition of 4-hydroxytamoxifen (OHT). (B) Centromere localization of the CENP-U-PHF8^ΔPHD^-Mer fusion after addition of OHT based on immunofluorescence with anti-estrogen receptor (ER) antibodies, which recognize Mer (red). Centromeres were marked by anti-CENP-A (green). The mutant version of CENP-U-PHF8-Mer (CENP-U-PHF8-DD-Mer) or CENP-U-Mer also localized to centromeres. CENP-A was used as a centromere marker. Scale bar, 10 μm. (C) H4K20me1 levels at CENP-A nucleosomes in DT40 cells expressing CENP-U-PHF8^ΔPHD^-Mer fusion before and after OHT addition based on western blot analysis. Centromeric H4K20me1 level was decreased in DT40 cells expressing CENP-U-PHF8^ΔPHD^-Mer, but not in DT40 cells expressing CENP-U-PHF8-DD-Mer or CENP-U-Mer. (D) Immunofluorescence of cells expressing CENP-U- PHF8^ΔPHD^-Mer with antibodies against various centromere proteins including CENP-A, CENP-C, CENP-H, and CENP-T. Scale bar, 10 μm. (E) Measurements of signal intensities for CENP-A, CENP-C, CENP-H, and CENP-T in DT40 cells expressing CENP-U-Mer, CENP-U-PHF8^ΔPHD^-Mer, or CENP-U-PHF8-DD-Mer. Results were plotted as the average of signal intensities in ten kinetochores per individual cell with each antibody (n > 7 cells). Error bars represent SD. Asterisk indicates statistically significance (Student’s t test).

To determine the functional significance of the H4K20me1 modification at centromeres, we devised an experimental system to eliminate centromeric H4K20me1 using chicken DT40 cells (Figure 4A). To do this, we prepared a fusion of CENP-U to the catalytic (Jumonji) domain (Figures S4A and S4B) of the H4K20me1 histone demethylase PHF8 (PHF8^ΔPHD^) (Liu et al., 2010; Qi et al., 2010). As CENP-U is a centromere protein and is associated with the CENP-A domain (Foltz et al., 2006; Minoshima et al., 2005), the CENP-U-PHF8^ΔPHD^ fusion should cause H4K20me1 to be demethylated specifically in CENP-A-associated chromatin (Figure 4A). In order to prevent the chimeric protein from acting constitutively, CENP-U-PHF8^ΔPHD^ was fused to a murine-estrogen-receptor (Mer)-tag (Shang et al., 2013). The Mer-tagged protein is normally restrained in the cytoplasm by HSP90 unless released from it by addition of 4-hydroxytamoxifen (OHT). Prior to addition of OHT, the CENP-U-PHF8^ΔPHD^-Mer fusion did not localize to centromeres. However, following OHT addition, we clearly detected centromere localization of the CENP-U-PHF8^ΔPHD^-Mer (Figures 4A and 4B).

The CENP-U-PHF8^ΔPHD^-Mer fusion is functional, as levels of H4K20me1 on CENP-A nucleosomes were dramatically reduced in cells expressing the CENP-U-PHF8^ΔPHD^-Mer fusion following OHT addition (Figure 4C). This reduction was not observed in cells expressing a mutant version of PHF8 (PHF8-DD) (Figure 4C) containing three point mutations in the Jumonji domain (Figure S4B). We note that PHF8 also catalyzes demethylation of other histone modifications, including H3K9me1/2 and H3K27me2. However, H4K20me1 levels are preferentially increased following PHF8 siRNA treatment (Liu et al., 2010; Qi et al., 2010), suggesting that PHF8 primarily demethylates H4K20me1. Here, we confirmed that levels of H3K9me2 in centromeric chromatin were not reduced following activation of PHF8 at centromeres (Figures S4C and S4D). Importantly, H3K9me1 and H3K27me2 were not enriched in the nonrepetitive centromeres of DT40 cells (Figure S1).

Given the availability of a system for controlled depletion of H4K20 monomethylation at centromeres, we examined the localization of other essential centromere proteins following depletion of centromeric H4K20me1. Although centromeric CENP-A and CENP-C signals were clearly detected in those cells, we observed a strong reduction in CENP-H and CENP-T localization to centromeres compared with control cells (Figures 4D and 4E). CENP-C and CENP-T function in parallel pathways for outer kinetochore assembly (Hori et al., 2013), and CENP-H localization occurs downstream of CENP-T. Consistent with the reduction in CENP-T and CENP-H levels, we found that many cells expressing the CENP-U-PHF8^ΔPHD^-Mer fusion were delayed at prometaphase and died, similar to CENP-T- or CENP-H-deficient cells (Hori et al., 2008; Okada et al., 2006).

We also examined H4K20me1 levels at CENP-A chromatin in various DT40 knockout cell lines for kinetochore/centromere proteins and found that H4K20me1 levels were reduced in HJURP-deficient cell lines, but not in CENP-C-, CENP-T-, and KNL1-deficient cell lines (Figures S4E and S4F). This suggest that H4K20me1 occurs downstream of CENP-A incorporation into centromeres and upstream of the CENP-T assembly pathway.

Together, these experiments suggest that H4K20me1 is required for kinetochore assembly by promoting CENP-T localization.

## Discussion

It has long been known that assembly of kinetochores requires an epigenetic component (Allshire and Karpen, 2008; Earnshaw and Migeon, 1985), but the chromatin modifications involved were mysterious. Whereas deposition of CENP-A at centromeric regions is crucial for centromere-specification, it was largely unknown whether additional epigenetic features are involved in centromere specification and/or kinetochore assembly. In addition, many recent studies have shown that a plethora of histone modifications enable chromatin to adopt a wide range of functional states, but these have yet to be mapped in centromeric chromatin because of the repetitive nature of the regions in most metazoans.

In this study, we demonstrated that H4K20 monomethylation occurs on centromeric CENP-A nucleosomes in both chicken and human cells, where it is required for kinetochore assembly. In centromere regions with highly repetitive sequences, such as human α-satellite, many histone modification markers for heterochromatin, including H3K9me3 or H4K20me3, are detected (Ruthenburg et al., 2007). However, these heterochromatin markers were not detected at chicken nonrepetitive centromeres (Cen5, 27, and Z) or at neocentromeres. Instead, H4K20me1 was specifically detected at both nonrepetitive and repetitive centromeres (Figures 1 and S1). We propose that centromeric H4K20me1 primarily occurs throughout the CENP-A-associated region and is distinct from flanking heterochromatin markers.

Our system for chromosome engineering and neocentromere induction (Shang et al., 2013) previously led us to suggest that CENP-A is essential for de novo centromere formation. Thus, even though kinetochore assembly can occur without CENP-A chromatin if CENP-T or CENP-C N termini are artificially tethered at a noncentromere locus (Gascoigne et al., 2011; Hori et al., 2013), our data suggest that establishment of a functional CENP-A chromatin domain is a crucial step for kinetochore assembly at natural centromeres. Ectopic low levels of CENP-A are located at noncentromere loci (Shang et al., 2013), but they are not functional, possibly because they lack H4K20me1 modification (Figure 2).

We hypothesize that once CENP-A nucleosomes acquire H4K20me1, the CENP-A chromatin is “matured” allowing CENP-T and its downstream proteins to assemble the functional kinetochore. Our cell-cycle analysis demonstrated that H4K20me1 is constitutively detected at centromeres throughout the cell cycle (Figure S2), and this modification does not occur before CENP-A is incorporated into centromeres. Therefore, “maturation of CENP-A nucleosomes” by the H4K20me1 modification must occur during G1 only after CENP-A deposition into centromeres to ensure faithful kinetochore assembly.

It remains unclear how H4K20me1 contributes to kinetochore assembly beyond being required to recruit CENP-T. Importantly, this modification is frequently linked with transcription, which has been shown to be important for kinetochore maintenance and assembly (Bergmann et al., 2011, 2012; Nakano et al., 2008). Thus, H4K20me1 might be linked to the regulation of centromeric transcription. Alternatively, or in addition, it may have a role in establishing the structure of centromeric chromatin, thereby facilitating kinetochore assembly, for example, by providing a preferred binding site for factors that may promote the CENP-T-W-S-X complex (Nishino et al., 2012) association with chromatin. The discovery of H4K20me1 in CENP-A nucleosomes provides a significant step toward understanding the epigenetic regulation of kinetochore assembly.

## Experimental Procedures

### Cell Culture

DT40 and HeLa cells were cultured by a standard method. Plasmid constructs were transfected with a Gene Pulser II electroporator (Bio-Rad) into DT40 cells as described previously (Hori et al., 2008).

### ChIP-Seq Analysis

For chromatin immunoprecipitation, we used our collection of antibodies for histone modifications (Kimura et al., 2008). Two kinds of monoclonal antibodies (15F11 and 22G3) against H4K20me1 were used. Chromatin isolation and immunoprecipitation were performed by the previous method (Shang et al., 2010, 2013). DNA was extracted from immunoprecipitates and was subjected into a HiSeq 2000 DNA sequencer (Illumina). Sequenced data were mapped into a Chicken Genome database (NCBI, Build 3.1) with the Burrows-Wheeler Aligner (BWA) (v. 0.6.1) program (Li and Durbin, 2009).

### Immunofluorescence

For immunofluorescence analysis of H4K20me1 in DT40 or HeLa, cells expressing CENP-A-GFP were treated with hypotonic buffer (20 mM Tris-HCl [pH 7.4], 1.5 mM KCl) at room temperature for 10 min and were cytospun into glass slides. The samples were fixed in cold methanol for 20 min at −20°C, following treatment of 4% paraformaldehyde for 20 s. Then the samples were treated with 0.5% Triton X-100 in PBS for 5 min, and Cy3-labeled mouse monoclonal antibodies against H4K20me1 (22G3) were added as primary antibodies. For immunofluorescence analysis of centromere proteins, we used a previously described method (Hori et al., 2013). Immunofluorescence images were collected with a Cool SNAP HQ camera (Roper Scientific Japan) mounted on an Olympus IX71-inverted microscope with a 100× objective lens, together with a filter wheel. We also used an N-SIM Super-Resolution microscope system (Nikon) with an EM CCD camera (Andor).

## Author Contributions

T.H. and W.-H.S. performed entire experiments and analyzed the data. A.T. and A.F. performed deep sequencing. S.M., N.M., and K.I. analyzed deep-sequencing data. O.M. performed the prSet7-tethering experiments. G.V. performed immunofluorescence experiments on chromatin fibers. H.K. prepared antibodies against various histone modifications. W.C.E. suggested some experiments and contributed to the preparation of the manuscript. T.H. and T.F. designed all experiments, and T.F. wrote the manuscript.

## Accession Numbers

The DNA Data Bank of Japan (DDBJ) accession number for the ChIP-seq data reported in this paper is DRA002224.

## Supplemental Information

Document S1. Figures S1–S4

Document S2. Article plus Supplemental Information
