# Supplementary material for: Histone H4 Lys 20 Monomethylation of the CENP-A Nucleosome Is Essential for Kinetochore Assembly
Source: Dev Cell. 2014 Jun 23;29(6):740–9. doi: 10.1016/j.devcel.2014.05.001 (PMC4081567; doi:10.1016/j.devcel.2014.05.001)
Supplement: Document S1. Figures S1–S4 [file mmc1.pdf]

Developmental Cell, Volume *29*

Supplemental Information

Histone H4 Lys 20 Monomethylation of the CENP-A Nucleosome Is Essential for Kinetochore Assembly

Tetsuya Hori, Wei-Hao Shang, Atsushi Toyoda, Sadahiko Misu, Norikazu Monma, Kazuho Ikeo, Oscar Molina, Giulia Vargiu, Asao Fujiyama, Hiroshi Kimura, William C. Earnshaw, and Tatsuo Fukagawa

Fig. S1

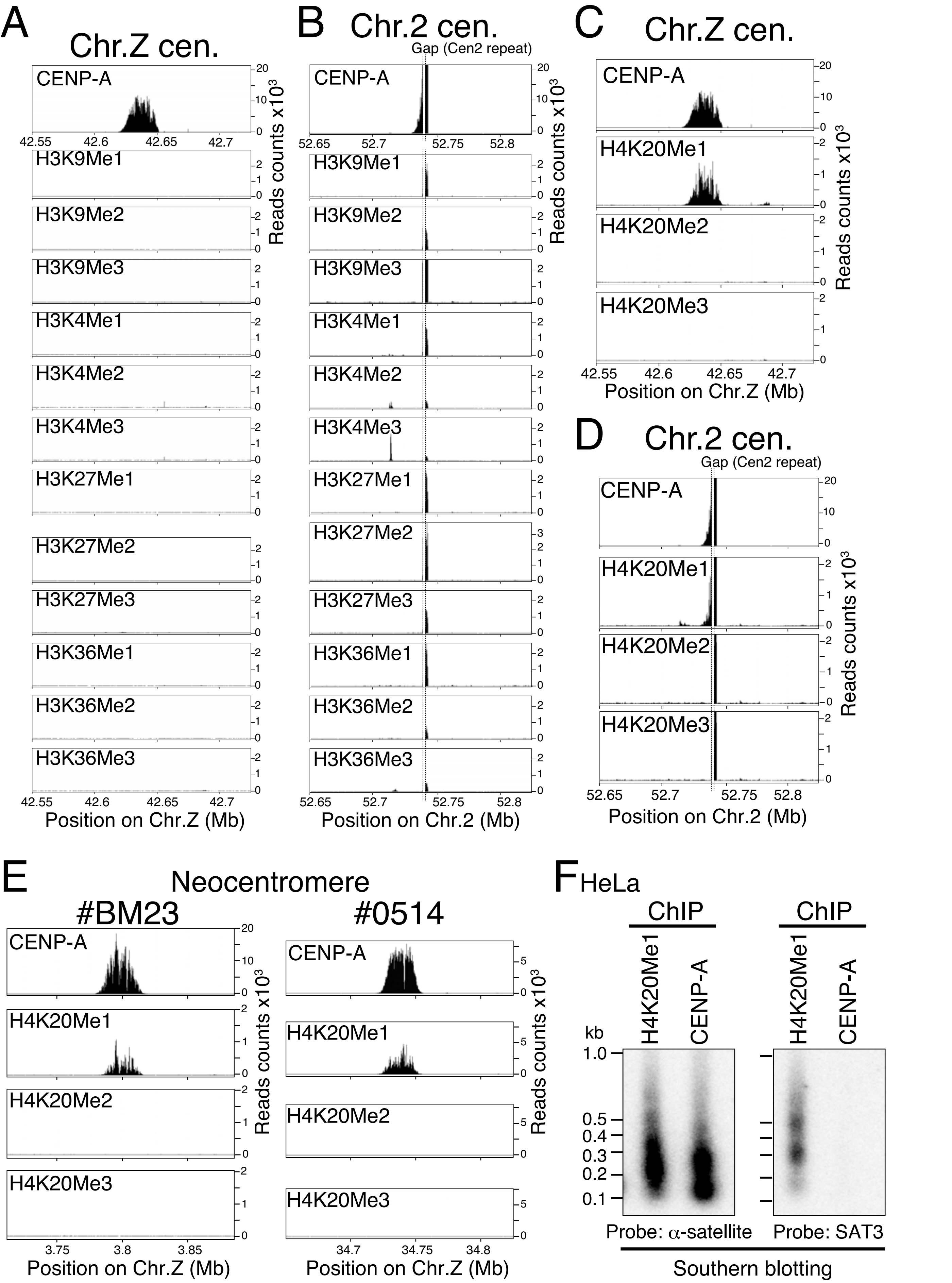
A
Chr.Z cen.
CENP-A
H3K9Me1
H3K9Me2
H3K9Me3
H3K4Me1
H3K4Me2
H3K4Me3
H3K27Me1
H3K27Me2
H3K27Me3
H3K36Me1
H3K36Me2
H3K36Me3
Reads counts x10^3
Position on Chr.Z (Mb)
B
Chr.2 cen.
Gap (Cen2 repeat)
Position on Chr.2 (Mb)
C
Chr.Z cen.
H4K20Me1
H4K20Me2
H4K20Me3
D
Chr.2 cen.
E
Neocentromere
#BM23
#0514
F
HeLa
ChIP
kb
Probe: α-satellite
Probe: SAT3
Southern blotting

Fig. S2

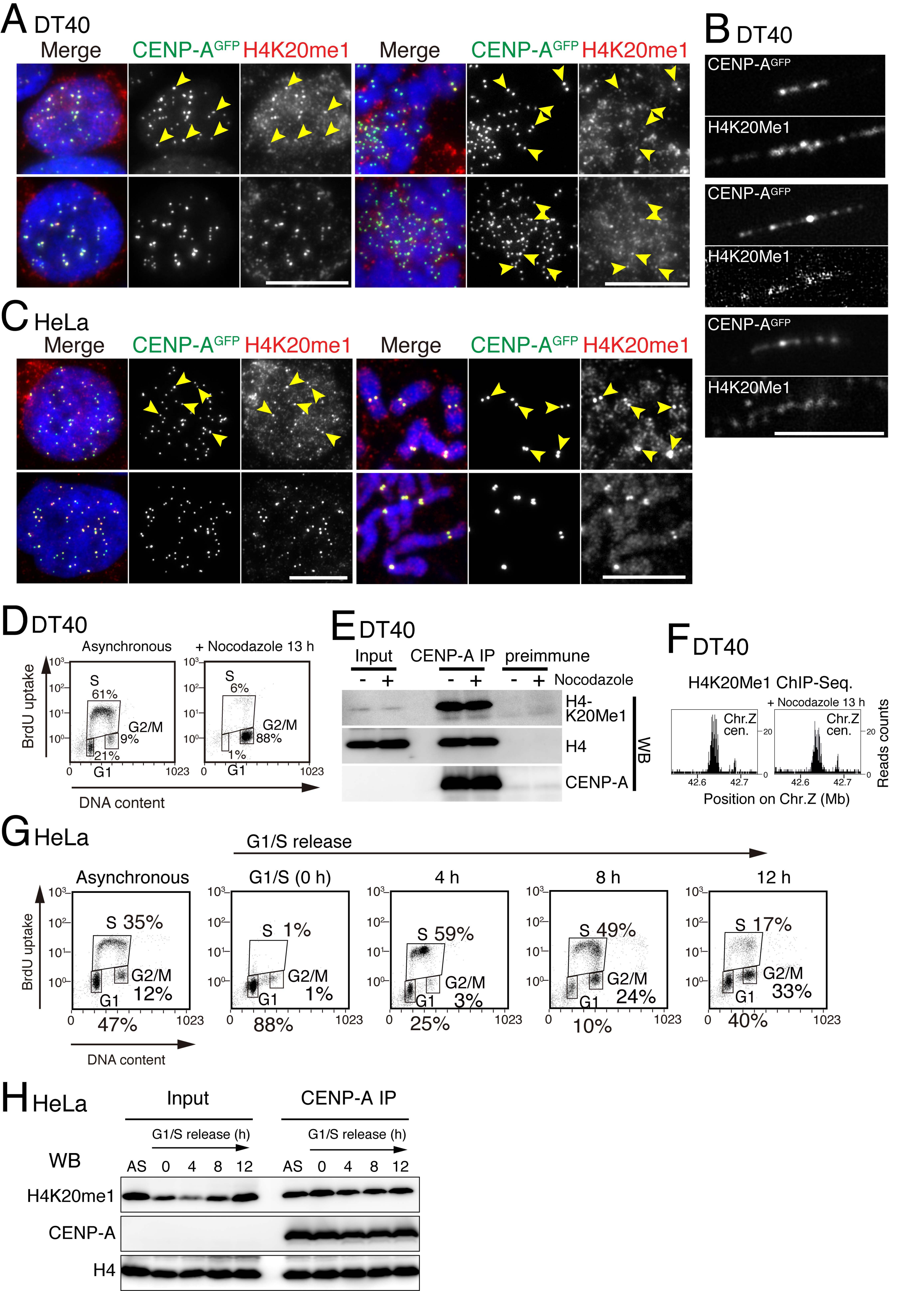
A DT40
Merge
CENP-A^{GFP} H4K20me1
B DT40
CENP-A^{GFP}
H4K20Me1
C HeLa
D DT40
Asynchronous
+ Nocodazole 13 h
BrdU uptake
S 61%
G2/M 9%
21%
G1
S 6%
G2/M 88%
1%
0
1023
DNA content
E DT40
Input
CENP-A IP
preimmune
- + - + - +
Nocodazole
H4-K20Me1
H4
CENP-A
WB
F DT40
H4K20Me1 ChIP-Seq.
+ Nocodazole 13 h
Chr.Z cen.
Reads counts
42.6
42.7
Position on Chr.Z (Mb)
G HeLa
G1/S release
Asynchronous
G1/S (0 h)
4 h
8 h
12 h
S 35%
G2/M 12%
47%
S 1%
G2/M 1%
88%
S 59%
G2/M 3%
25%
S 49%
G2/M 24%
10%
S 17%
G2/M 33%
40%
H HeLa
Input
CENP-A IP
G1/S release (h)
WB
AS 0 4 8 12
H4K20me1
CENP-A
H4

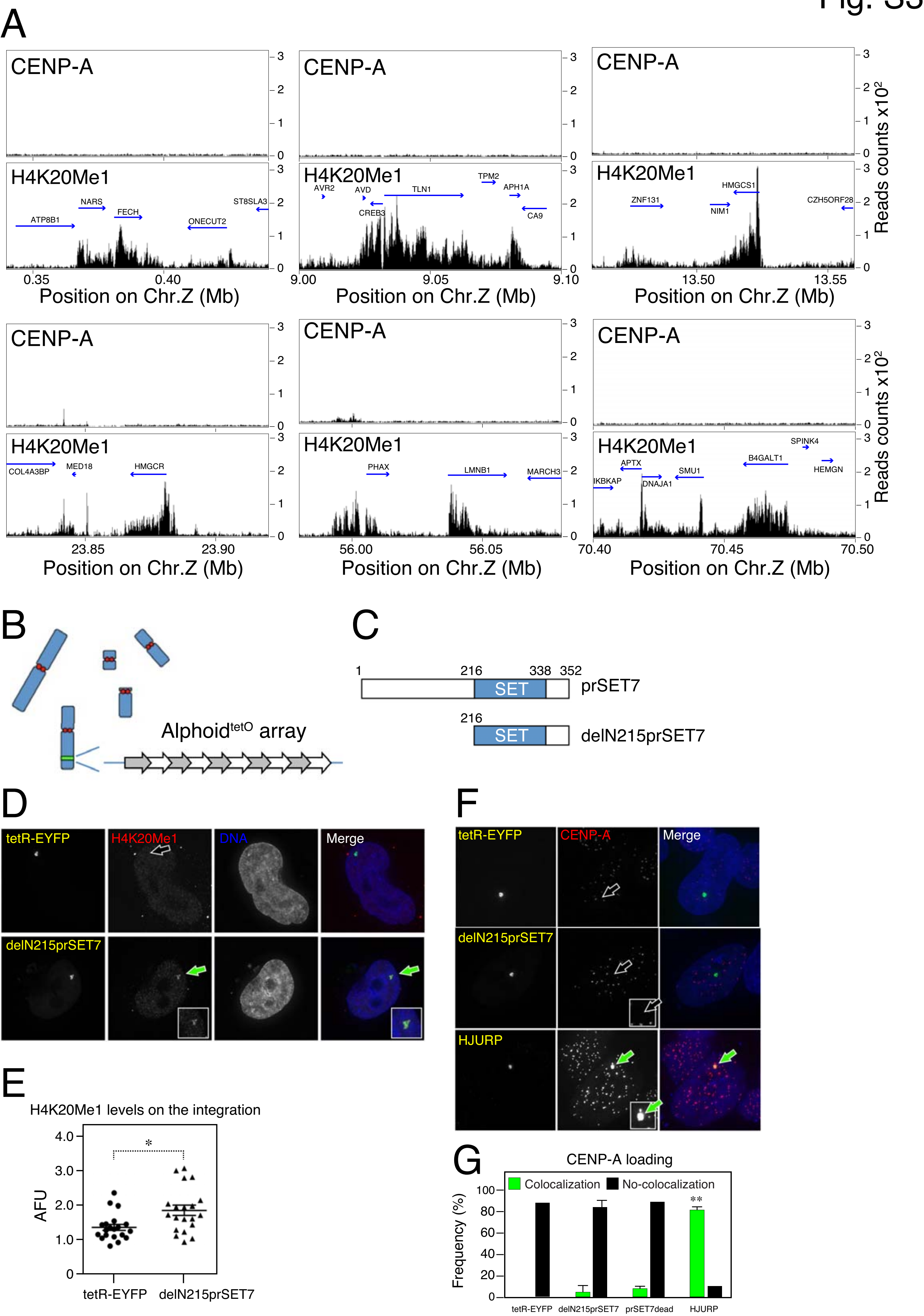
A
CENP-A
H4K20Me1
Position on Chr.Z (Mb)
Reads counts x10^2
ATP8B1
NARS
FECH
ONECUT2
ST8SLA3
AVR2
AVD
CREB3
TLN1
TPM2
APH1A
CA9
ZNF131
NIM1
HMGCS1
CZH5ORF28
COL4A3BP
MED18
HMGCR
PHAX
LMNB1
MARCH3
IKBKAP
APTX
DNAJA1
SMU1
B4GALT1
SPINK4
HEMGN
B
Alphoid^tetO array
C
1
216
338
352
SET
prSET7
216
SET
delN215prSET7
D
tetR-EYFP
H4K20Me1
DNA
Merge
delN215prSET7
E
H4K20Me1 levels on the integration
AFU
tetR-EYFP
delN215prSET7
F
tetR-EYFP
CENP-A
Merge
delN215prSET7
HJURP
G
CENP-A loading
Colocalization
No-colocalization
Frequency (%)
tetR-EYFP
delN215prSET7
prSET7dead
HJURP

Fig. S4

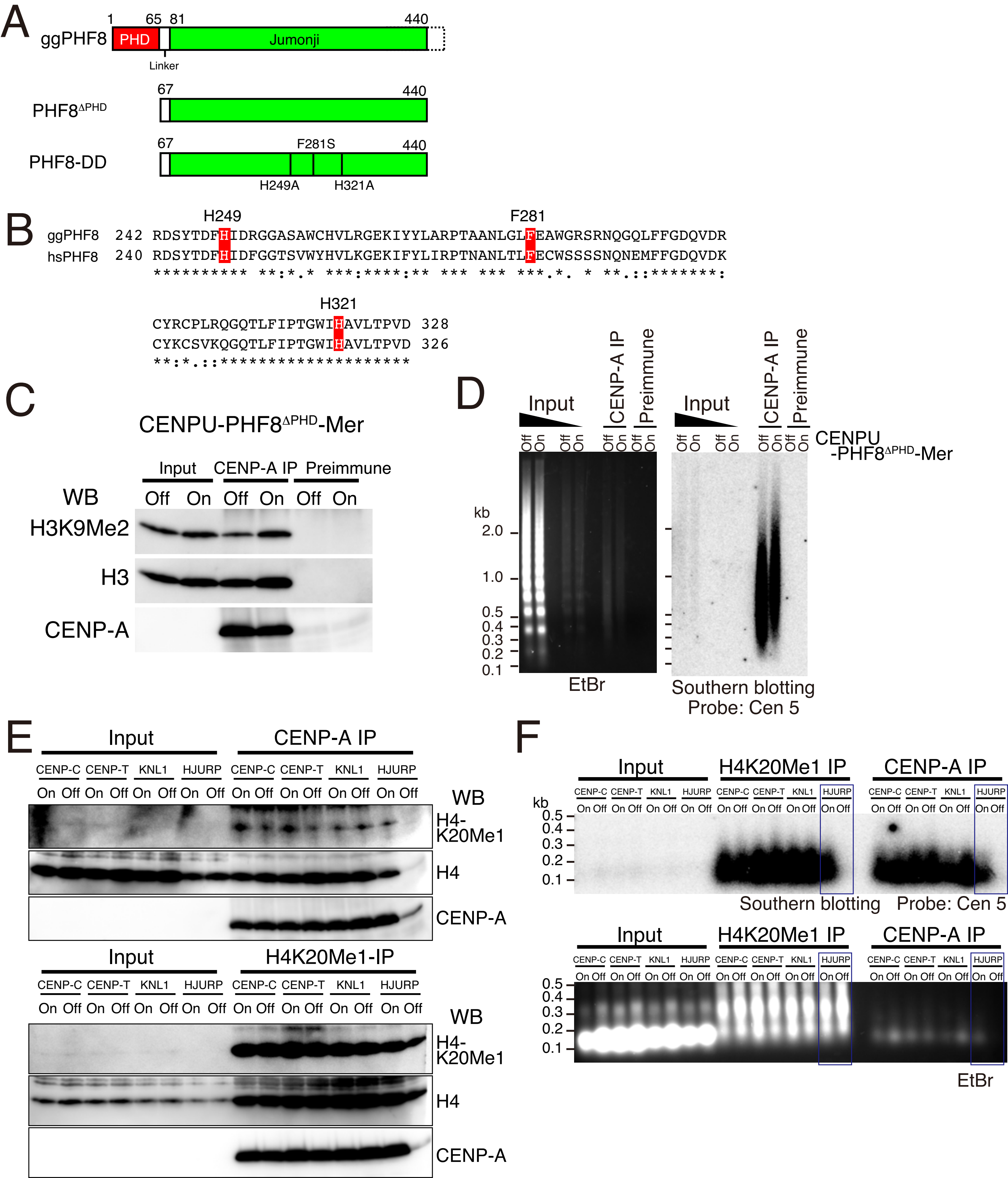
A
ggPHF8
1
65
81
440
PHD
Jumonji
Linker
PHF8ΔPHD
67
440
PHF8-DD
67
F281S
440
H249A
H321A
B
H249
F281
ggPHF8 242 RDSYTDFHIDRGGASAWCHVLRGEKIYYLARPTAANLGLFEAWGRSRNQGQLFFGDQVDR
hsPHF8 240 RDSYTDFHIDFGGTSVWYHVLKGEKIFYLIRPTNANLTLFECWSSSSNQNEMFFGDQVDK
********** **:*.* ***:****:** *** *** ***.*. * **.::*******:
H321
CYRCPLRQGQTLFIPTGWIHAVLTPVD 328
CYKCSVKQGQTLFIPTGWIHAVLTPVD 326
:*.::******************
C
CENPU-PHF8ΔPHD-Mer
Input
CENP-A IP
Preimmune
WB
Off On Off On Off On
H3K9Me2
H3
CENP-A
D
Input
CENP-A IP
Preimmune
Input
CENP-A IP
Preimmune
Off On Off On Off On Off On
Off On Off On Off On Off On
CENPU
-PHF8ΔPHD-Mer
kb
2.0
1.0
0.5
0.4
0.3
0.2
0.1
EtBr
Southern blotting
Probe: Cen 5
E
Input
CENP-A IP
CENP-C CENP-T KNL1 HJURP CENP-C CENP-T KNL1 HJURP
On Off On Off On Off On Off On Off On Off On Off On Off
WB
H4-K20Me1
H4
CENP-A
Input
H4K20Me1-IP
CENP-C CENP-T KNL1 HJURP CENP-C CENP-T KNL1 HJURP
On Off On Off On Off On Off On Off On Off On Off On Off
WB
H4-K20Me1
H4
CENP-A
F
Input
H4K20Me1 IP
CENP-A IP
CENP-C CENP-T KNL1 HJURP CENP-C CENP-T KNL1 HJURP CENP-C CENP-T KNL1 HJURP
On Off On Off On Off On Off On Off On Off On Off On Off On Off On Off On Off On Off
kb
0.5
0.4
0.3
0.2
0.1
Southern blotting
Probe: Cen 5
Input
H4K20Me1 IP
CENP-A IP
CENP-C CENP-T KNL1 HJURP CENP-C CENP-T KNL1 HJURP CENP-C CENP-T KNL1 HJURP
On Off On Off On Off On Off On Off On Off On Off On Off On Off On Off On Off On Off
0.5
0.4
0.3
0.2
0.1
EtBr

Legends for Supplementary Figures

Figure S1. ChIP-seq profiles for various histone modifications around repetitive or non-repetitive centromeres, related to Figure 1

(A) High-resolution ChIP-seq profiles around centromere region on chromosome Z (non-repetitive centromere) with anti-CENP-A, -H3K9me1, -H3K9me2, -H3K9me3, -H3K4me1, -H3K4me2, -H3K4me3, -H3K27me1, -H3K27me2, -H3K27me3, -H3K36me1, -H3K36me2, and -H3K36me3 antibodies.

(B) High-resolution ChIP-seq profiles around centromere region on chromosome 2 (repetitive centromere) with anti-CENP-A, -H3K9me1, -H3K9me2, -H3K9me3, -H3K4me1, -H3K4me2, -H3K4me3, -H3K27me1, -H3K27me2, -H3K27me3, -H3K36me1, -H3K36me2, and -H3K36me3 antibodies.

(C) High-resolution ChIP-seq profiles around centromere region on chromosome Z with anti-CENP-A, -H4K20me1, -H4K20me2, and -H4K20me3 antibodies.

(D) High-resolution ChIP-seq profiles around centromere region on chromosome 2 with anti-CENP-A, -H4K20me1, -H4K20me2, and -H4K20me3 antibodies.

(E) High-resolution ChIP-seq profiles around neocentromeres (in #BM23 or #0514 cells) with anti-CENP-A, -H4K20me1, -H4K20me2, and -H4K20 me3 antibodies.

(F) Chromatin fraction was prepared from human HeLa cells and immunoprecipitation was performed with anti-H4K20me1 or -CENP-A antibodies. DNAs were recovered from immunoprecipitates and applied for Southern hybridization with α-satellite or sat3 as a probe. Both DNAs were hybridized with the α-satellite probe, which is a major component of human centromeres.

Figure S2. H4K20me1 at centromeres constitutively occurs across the cell-cycle, related to Figure 1

(A) Representative various images for co-localization of H4K20me1 with CENP-A-GFP in interphase and mitotic DT40 cells. Arrows show co-localization. As accessibility of H4K20me1 to target sites is not efficient, background signals were detected in some images. Bar, 10 μm.

(B) Co-localization of H4K20me1 with CENP-A on chromatin fibers from DT40 cells. Three examples are shown. Bar, 5 μm.

(C) Representative various images for co-localization of H4K20me1 with CENP-A-GFP in interphase and mitotic HeLa cells. Arrows show co-localization. As observed in DT40 cells, background signals were detected in some images. Bar, 10 μm.

(D) FACS profiles of asynchronous and mitotic DT40 cells. Mitotic DT40 cells were prepared by treating cells with nocodazole for 13 h. 88% of cells were in G2/M fraction based on FACS analysis.

(E) Western blot analysis of CENP-A chromatin with anti-H4K20me1, -panH4, and -CENP-A antibodies from asynchronous or mitotic DT40 cells. Levels of H4K20me1 at CENP-A chromatin from asynchronous cells are similar to those from mitotic cells.

(F) ChIP-seq analysis of Cen Z region from asynchronous or mitotic DT40 cells with anti-H4K20me1 antibodies. ChIP-seq profile from mitotic cells is similar to that from asynchronous cells.

(G) Synchronization of HeLa cells by release from double thymidine block. Time 0 indicates G1/S boundary. We collected cells at 0, 4, 8, and 12 h time points and examined H4K20me1 levels in CENP-A chromatin based on IP-Western experiments (see H).

(H) Western blot analysis of CENP-A chromatin with anit-H4K20me1, -CENP-A and -panH4 antibodies from various cell-cycle stages in HeLa cells. Whereas total H4K20me1 levels were decreased at 4 h (during S phase: see input), H4K20me1 levels at CENP-A chromatin are constant across the cell-cycle, suggesting that H4K20me1 at CENP-A chromatin constitutively occurs.

Figure S3. H4K20me1 in non-centromeric region does not induce centromere formation, related to Figure 2

(A) ChIP-seq profiles for CENP-A and H4K20me1 in non-centromeric region. Regions with high enrichment for H4K20me1 were selected and CENP-A profiles were also shown. H4K20me1 is highly enriched in gene bodies at non-centromere loci, but CENP-A was not accumulated in these loci. Some transcribed genes are also shown.

(B) HeLa 3-8 cell line containing an α-satellite (alphoid) DNA array integration with tetO sites (alphoid^{tetO} array) in a chromosome arm.

(C) Lengths of prSET7 protein fused to tetR-EYFP. These tetR fusions were targeted to an alphoid^{tetO} array in HeLa cells shown in (B).

(D) H4K20me1 IF analysis of interphase HeLa 3-8 cells expressing tetR-EYFP, and tetR-EYFP-delN215prSET7 constructs to confirm ectopic deposition of H4K20me1 on the alphoid^{tetO} sites in a chromosome arm.

(E) H4K20me1 signal quantification after the expression of the indicated tetR-EYFP fusion proteins in HeLa 3-8 cells (Mann-Whitney test; * $p<0.05$).

(F) Representative images of tetR-EYFP fusion proteins with the chromatin modifiers indicated tethered on the alphoid-DNA array integration and IF staining for CENP-A.

(G) Quantification of the frequency of CENP-A loading by the different tetR-EYFP fusion proteins on the integration in HeLa3-8 cell line. Results were plotted as the average of two independent IF experiments. Error bars represent the standard deviation (SD) (Chi-square test; * $p<0.05$ and ** $p<0.01$).

Figure S4. Targeting of chicken PHF8 to centromeres causes defects of kinetochore assembly, related to Figure 4

(A) Diagram chicken full-length PHF8, PHF8$^{\Delta PHD}$ that contains catalytic domain (Jumonji) and PHF8-DD that has mutations in the catalytic domain.

(B) Mutation sites of PHF8-DD. Amino acids sequence of chicken (ggPHF8) and human PHF8 (hsPHF8) are aligned. Identical amino acids are indicated with * and similar amino acids are indicated with : or ·.

(C) Levels of H3K9me2 at centromeric chromatin in cells expressing CENP-U-PHF8$^{\Delta PHD}$-Mer in the absence (Off) or presence (On) of OHT.

(D) Analysis of centromereic DNA used in (C). Centromeric DNAs were recovered by IP with anti-CENP-A from chromatin fraction partially digested with MNase. These DNAs were hybridized with a Cen5 (centromere 5) probe.

(E) Western blot analysis of CENP-A chromatin with anti-H4K20me1, -panH4 and -CENP-A antibodies in various DT40 knockout cell lines for centromere/kinetochore proteins including CENP-C, CENP-T, KNL1, and HJURP. H4K20me1-associated chromatin in these cell lines was also analyzed. As these lines are conditional knockout cell lines, expression of each protein can be turned off by addition of tetracycline (Off), In the absence of tetracycline, these cells behave as wild-type cells (On). HJURP is a CENP-A specific chaperon and both of CENP-A and H4K20me1 levels were decreased in the CENP-A immunoprecipitates following HJURP disruption. CENP-A and H4K20me1 levels in CENP-A chromatin were not changed in knockout cell lines for CENP-C, CENP-T, and KNL1.

(F) H4K20me1- or CENP-A-associated DNAs were recovered from various DT40 knockout cell lines for centromere/kinetochore proteins including CENP-C, CENP-T, KNL1, and HJURP. Southern blot analysis of these DNAs using Cen5 DNA as a probe. The centromere DNAs hybridized with the Cen5 probe were decreased both in the H4K20me1 and CENP-A immunoprecipitates following HJURP disruption.
